# Oral Presentations - Abstracts of the 27^th^ Annual Congress of the SBRA. Aracaju/SE - Brazil, 2023

**DOI:** 10.5935/1518-0557.20230070

**Published:** 2024

**O-01. Prediction of Varicocelectomy Outcomes Using 1 H NMR-based Metabonomics Assays of Blood Serum**

**Filipe Tenorio Lira Neto^1^, Estefane Cunha Santos Salles^2^, Ronmilson Alves Marques^2^, João Eduardo Freire Fonte^3^, Salvador Vilar Correia Lima^2^, Ricardo Oliveira^2^**

*^1^ IMIP - Recife - PE - Brasil*

*^2^ UFPE - Recife - PE - Brasil*

*^3^ Real Hospital de Beneficência Portuguesa - Recife - PE - Brasil*

**Objective:** To create metabolomics models capable of segregating men who improved semen analysis (SA) parameters after microsurgical varicocelectomy (MV) from those who did not, using Hydrogen-1 nuclear magnetic resonance (1H NMR) spectra of blood serum from of preoperative samples.

**Methods:** We recruited 32 infertile men with palpable varicocele and abnormal semen analysis (SA). Preoperative peripheral venous blood samples were centrifuged at 3000 g for 15 minutes to separate the cellular component from the supernatant serum. The blood serum samples were kept frozen at -40°C until analysis. 1H NMR spectra of blood serum were obtained and used to create metabonomics models. All participants were treated with artery and lymphatic sparing MV by the same surgeon. Improvement was defined as an increase of at least 20% in the total motile progressive sperm count (TMC) of the post-operative SA when compared to the baseline.

**Results:** Eighteen (56%) participants had SA improvement after MV and were included in the improved (I) group, the remaining 14 participants were included in the non-improved (NI) grop. There were no differences regarding the baseline characteristics and SA parameters. The I group had lower baseline luteinizing hormone levels when compared to the NI group (4.3 vs 7.0 mUI/mL), and there were no other differences concerning the baseline sexual hormones levels. The I group showed higher post-operative sperm concentration, total sperm count, progressive motility, TMC, and morphology than the group non-improvement group (Table 1). Concerning the changes of TMC from baseline, the I group had a median increase of 10.6 x 106 sperm whereas the group NI had a median decrease of -5.1 x 106 sperm (*P*-value < 0.05). When the changes were analyzed using percentage, this difference was further highlighted, the group I demonstrated an increase of 223% (range 44% to 34000%) and the group NI had a decrease of -59% (range -9% to - 100%) (*P*-value < 0.05).Using Linear Discriminant Analysis (LDA), we created a model that discriminated the men who improved SA from those who did not with sensitivity of 88.2%, specificity of 80.0% and accuracy of 84.4% after leave-one-out cross validation. We identified 4 metabolites that were important for group segregation: low density lipoprotein, isoleucine, glutamate, and methylamine.

**Conclusion:** We described the use of metabonomics model to predict the outcomes of MV in infertile men with varicocele with high accuracy. The most important metabolites for groups segregation are involved in amino acid metabolism and oxidative stress response, highlighting the pivotal role of these mechanisms in the pathophysiology of varicocele. These models may help counseling infertile men with varicocele regarding their prognosis after surgery.

**O-02. Day 6 Euploid Embryos vs. Day 5 Euploid Embryos: How important is morphology in selecting the embryo to transfer?**

**Carlos Augusto Zarate Nissel^1^, Patricia Flórido^1^, Mayra Satiko Lemos Nakano^2^, Alecsandra Prado Gomes^2^, Hamilton de Martin^2^, Mariana Gomes Fujii^2^, Tatiana Carvalho Souza Bonetti^1^, Pedro Augusto Araújo Monteleone^2^**

*^1^ Disciplina de Ginecologia - Departamento de Obstetrícia e Ginecologia, Faculdade de Medicina da Universidade de São Paulo - São Paulo - SP - Brasil*

*^2^ Centro de Reprodução Humana Monteleone - São Paulo - SP - Brasil*

**Objective:** Several studies have shown that live birth rates (LBR) of day 5 (D5) blastocyst transfers are significantly higher when compared with day six (D6) embryos in both fresh and vitrified-warmed embryo transfer cycles. Despite the slower development of D6 blastocyst and lower pregnancy rates, they still have a reasonable implantation potential and, therefore, they are regularly transferred. Most studies evaluating results of D5 and D6 blastocyst transfer did not considered Preimplantation Genetic Testing for Aneuploidy (PGT-A) and differences between transfer of euploid blastocysts on D5 versus D6 remains unclear. The objective of this study was to compare ongoing pregnancy rates (OPR) in in-vitro fertilization (IVF) cycles between euploid embryos that reached expanded blastocyst stage and were biopsied at the 5^th^ day of development with those reaching that stage at the 6^th^ day.

**Methods:** The retrospective study included single center real-world data from IVF cycles performed between 2019 and 2022, in which embryo biopsy for PGT-A was performed and euploid embryos were available for transfer. All embryos were cultured in a time-lapse incubator and were biopsied when they reached the expanded blastocyst stage, on D5 or D6 of development. Blastocysts were vitrified after biopsy and single euploid blastocyst transfers were placed in subsequent hormone replacement cycles.

**Results:** A total of 265 single blastocysts transfers were included, comprising 210 D5-embryos and 55 D6-embryos. Groups have similar mean women age (37.6±3.2 versus 38.4±3.4; *p*=0.122), and most transfers were from first IVF cycles (D5: 75% and D6: 62%; *p*=0.055). Pregnancy rates were similar between groups (D5: 56% and D6: 49%; *p*=0.346), but OPR was higher on D5 group (37%) compared to D6 group (24%; *p*=0.065) with borderline statistical significance. Nevertheless, the incidence of top-quality (TQ) blastocysts transferred on D5 (81.4%) was significantly higher than those in D6 (49.1%; *p*<0.001). Then, a multivariate logistic regression model was performed, the OPR was associated to TQ-blastocysts transferred (OR: 2.66; *p*=0.005) independently from the day of blastocyst development (OR: 0.66; *p*=0.258) or women age (OR: 1.04; *p*=0.339). To assess the interaction between TQ and day of development on OPR, a generalized linear model with a binomial distribution was conducted. The model showed a significant association of TQ and OPR (*p*=0.024), but not for day of development (*p*=0.190) or interaction of them (*p*=0.598). To confirm those findings, transfers were divided in four groups by quality of blastocyst transferred (TQ or noTQ) and day of development (D5 or D6). OPR rates were: TQ-D5 40.4%, noTQ-D5 25.6%, TQ-D6 33.3% and noTQ-D6 14.3%. There was a statistically significant difference between TQ-D5 vs. noTQ-D6: *p*=0.008, and borderline statistical difference between TQ-D5 vs. noTQ-D5: *p*=0.087 and TQ-D6 *vs*. noTQ-D6: *p*=0.096.

**Conclusion:** Our study indicates a remarkable disparity on OPR between euploid D5 and D6 embryos when morphology is not considered. However, the complete analysis showed a stronger relationship to the quality of the blastocyst transferred, thus morphology may be a better predictor of OPR than the day of development of euploid blastocysts. Limitations are the small number of euploid D6 blastocysts available for inclusion and its retrospective nature, but these findings bring an important discussion about the choice of euploid blastocysts of different morphology and development day. The doubt about transfer an euploid TQ-D6 embryo or a noTQ-D5 require further investigation to be completely elucidated, so does the underlying causes for this differences. Conduct more comprehensive investigations to thoroughly examine the factors contributing to the observed variations in success rates between euploid blastocysts transferred at the same development stage but with distinct developmental times and morphological classifications.

**O-03. Autologous Platelet-Rich Plasma for Endometrial Regeneration in Patients undergoing IVF Treatment with Thin Endometrium - a Prospective, Interventional Cohort Study**

Fernanda de Paula Rodrigues Neri^1^, Luciene K Tsukuda^1^, Gisele Venâncio Rodrigues^1^, Juliana Nabeiro Pereira^1^, Kerolyn Farias^1^, Mauricio Barbour Chehin^1^, Adriana Luckow Invitti^2^, Luiz Augusto Silva Nani^3^, Flavia Roche Moreira Latini^3^, Afonso José Pereira Cortez^3^, Manoel João Bastista Castello Girão^2^*, Aline Rodrigues Lorenzon^1^, Eduardo Leme Alves Motta^1^

*^1^ Huntington Medicina Reprodutiva - Eugin Group - São Paulo - SP - Brasil*

*^2^ Departamento de Ginecologia - EPM/UNIFESP - São Paulo - SP - Brasil*

*^3^ Colsan - São Paulo - SP - Brasil*

^*^ In Memoriam

**Objective:** A synchronized crosstalk between a receptive endometrium and a viable embryo is crucial to achieve a successful pregnancy. Endometrial thickness is considered ideal if ≥ 7mm triple-line at late follicular phase. Several strategies to improve refractory endometrium have been applied with lack of proven efficacy resulting in cycle’s cancelation. Autologous Platelet-Rich Plasma (PRP) is widely used for tissue regeneration by several areas of medicine, due to the fact that platelets are rich in numerous growth factors such as VEGF, TGF-b, PDGF, IGF1, EGF, among others, with the potential to promote tissue regeneration, proliferation and neoangiogenesis. The primary objective of this study was to evaluate the efficacy of PRP to improve endometrial thickness, volume and flow in patients with thin endometrium.

**Methods:** Prospective, interventional cohort study, conducted between August/2019-April/2022, including 78 patients undergoing endometrial preparation for Frozen Embryo Transfer, with a history of endometrial thickness <7mm at late follicular phase and hysteroscopy showing normal cavity anatomy. For endometrial preparation, oral estradiol valerate 8 mg/day were used. The autologous preparation of PRP was performed in a blood bank facility (COLSAN) encompassing 2 intrauterine infusion of 1.0 ml between cycle-days 7-10 and 11-14. The protocol was standardized and samples contained a mean of 2753,58±336,43/mm3 platelets. Endometrial thickness, volume and flow were measured by the same professional using a 3D Doppler velocimetry US before the 1st PRP and 2nd infusion and after 48-72h of 2nd infusion. Embryo transfer was performed considering endometrial parameters and hormone levels. All patients signed the informed consent form and the study was approved in the CEP/CONEP System. Statistical tests were performed accordingly and a *p*<0.05 was considered significant.

**Results:** Seventy-eight patients performed 110 cycles of endometrial preparation for PRP infusion. Endometrial thickness mean before 1st PRP infusion was 4,84±1,01mm and after 1st and 2nd infusion 5.59±1.04 and 5.99±1.09mm (*p*<0.0001). Endometrial volume mean before 1st PRP infusion was 0.70±0,36cc and 0.80±0.34 and 0.87±0.33 after 1st and 2nd infusion (*p*<0.0001). Endometrial flow was evaluated according to Applebaum classification (vascularity zones 1, 2, 3 and 4). The majority of patients had absent flow pre-PRP infusion (43.9%) and after 1st and 2nd infusions were classified in zone 2 (35.4% and 40.2%, *p*<0.0001). Sixty-seven patients (85,9%) proceed to 79 embryo transfer (ET) cycles. Endometrial thickness and volume were significant different after 1st and 2nd PRP infusions between patients that underwent ET or not (5.74±0.97 versus 5.18±1.10 and 6.24±0.92 versus 5.34±1.25mm, *p*=0.01 and 0.002; 0.80±0.34 versus 0.66±0.24 and 0.87±0.33 versus 0.68±0.29cc, *p*=0.03 and 0.008 respectively). Endometrial flow in the majority of patients without an ET were classified as absent flow after 2nd PRP infusion (45.2%), while the majority of patients with ET were classified in zone 2 (40.2%, *p*=0.0003). Mean maternal age was 38.9±4.5 years old, with ET 39.2±4.6 and without ET 38.1±4.1 (*p*=0.36). Positive pregnancy test (PPT) were achieved by 44.3% (35/79) of patients. Clinical pregnancy (CP/total ET) was 27.8%, miscarriage (miscarriage/PPT) 25.7%, biochemical pregnancy (BP/PPT) 11.4% and live birth rate (LB/total ET) 26.6%. Oocyte’s origin (donation or autologous), euploid embryo transfer and laboratory variables were similar between patients with a CP and negative PPT. The most prevalent obstetrical complication was preeclampsia (45.45%), and was similar in autologous and donated oocytes (50% versus 40%, *p*=0.69).

**Conclusion:** The use of a standard protocol of PRP with a rich concentration of platelets, was effective in improving endometrial thickness, volume and flow for patients with thin endometrium. The majority of patients (86%) were able to proceed to ET and the live birth rate (26.6%), when compared with the rates reported in the literature of patients with thin endometrium without PRP (19.44%), can be considered successful.

**O-4. Description of the creation of a training and evaluation of the perception of the multidisciplinary team of an assisted human reproduction center, for the reception of plural families**

**Queila Silva Raimundo^1^, Andreia Pegoraro^1^, Juliana Roberto Santos^1^, Renato Oliveira^1^, Caio Parente Barbosa^1^**

*^1^ Ideia Fertil - Santo Andre - SP - Brasil*

**Objective:** To describe the development process and the perception of understanding of the multidisciplinary team to welcome plural families, compatible with the needs and particularities inherent to this public. The possibility of treatment for plural families from the CFM 2015 resolution, led to an increase in demand for assisted human reproduction treatments, motivating the need for a better understanding of specialized centers, aiming at better reception. In this process, the entire team needs adaptation within the perspective of a more humane treatment.

**Methods:** Descriptive and exploratory study carried out at Instituto Ideia Fértil between August and December 2022. Initially, a multidisciplinary team was assembled with a doctor, nurse, psychologist and social worker to define the strategy and, later, the marketing team was included. First stage: meeting with all employees, both at department meetings and at CIPA lectures, addressing topics such as definitions of sex, gender and sexual orientation, as well as the importance of a welcoming environment, reflections on vulnerability and the importance of using the social name - if necessary - inclusion of the partner, if relevant, and approach to concepts on reproductive strategies. Theoretical classes and simulated situations were used. Additionally, during the training, a questionnaire was sent via Google forms with ten questions, in order to determine the employees' perception on the subject, the training. Among the questions, the following stand out: Do you know what all the letters in the acronym LGTBQIAPN+ mean?; Do you know the difference between gender identity and sexual orientation?; From 0 to 10, with 0 being none at all and 10 being a lot, how prepared do you feel to assist an LGBTQIAPN+ person?; Which of the factors below most arouse insecurity in the care of LGBTQIAPN+ people?; Are you aware of the social, economic, legal and psychological vulnerability faced by this population?; Do you believe that lectures, flyers and meetings would help you to serve this community?

**Results:** Of the 132 employees, 81 (61.3%) answered the questions. It is noteworthy that 55.6% knew the letters of the acronym LGBTQIAPN+; 92.6% considered it important for the Institute to promote these actions, with quarterly frequency for 35.8% and monthly for 34.6%; knew the difference between gender identity and sexual orientation 69 (85.2%); 59.3% knew which aspects related to gender identity and sexual orientation could influence care; on a scale of 0-10, 24.7% considered 9-10 for preparation in attendance; using incorrect pronouns and asking embarrassing questions were the main fear associated with care for 96.1%; 60.5% have knowledge about aspects of vulnerability; exchanging experience with experienced professionals would be the best way to assist in the service for 64.2% and knowledge on this topic generates a greater impact on citizenship for 80.2%.

**Conclusion:** Multidisciplinary training with expository classes and simulated situations for the team of Assisted Human Reproduction Centers in order to provide an affective approach to plural families is fundamental for the current needs and demands of society. This is an essential commitment to humanized medicine. The strategy of bringing professionals with experience was well accepted by the majority of the team, who understand this mainly as an aspect of citizenship. The main fears of professionals are related to the wrong use of pronouns and embarrassing questions. However, quarterly training can be an effective strategy. Increasing employee adherence and seeking continuous training are goals of the Institute that carried out this research.

